# Antiviral Responses in Cancer: Boosting Antitumor Immunity Through Activation of Interferon Pathway in the Tumor Microenvironment

**DOI:** 10.3389/fimmu.2021.782852

**Published:** 2021-12-02

**Authors:** Glauco Akelinghton Freire Vitiello, Wallax Augusto Silva Ferreira, Vladmir Cláudio Cordeiro de Lima, Tiago da Silva Medina

**Affiliations:** ^1^ Translational Immuno-Oncology Group, International Research Center, A.C. Camargo Cancer Center, São Paulo, Brazil; ^2^ Laboratory of Cytogenomics and Environmental Mutagenesis, Environment Section (SAMAM), Evandro Chagas Institute, Ananindeua, Brazil; ^3^ Department of Clinical Oncology, A.C. Camargo Cancer Center, São Paulo, Brazil; ^4^ National Institute of Science and Technology in Oncogenomics and Therapeutic Innovation, São Paulo, Brazil

**Keywords:** antiviral immune response, antitumor immunity, oncolytic viruses, interferons, epigenetic regulation, endogenous retroviral elements, immunotherapy

## Abstract

In recent years, it became apparent that cancers either associated with viral infections or aberrantly expressing endogenous retroviral elements (EREs) are more immunogenic, exhibiting an intense intra-tumor immune cell infiltration characterized by a robust cytolytic apparatus. On the other hand, epigenetic regulation of EREs is crucial to maintain steady-state conditions and cell homeostasis. In line with this, epigenetic disruptions within steady-state cells can lead to cancer development and trigger the release of EREs into the cytoplasmic compartment. As such, detection of viral molecules by intracellular innate immune sensors leads to the production of type I and type III interferons that act to induce an antiviral state, thus restraining viral replication. This knowledge has recently gained momentum due to the possibility of triggering intratumoral activation of interferon responses, which could be used as an adjuvant to elicit strong anti-tumor immune responses that ultimately lead to a cascade of cytokine production. Accordingly, several therapeutic approaches are currently being tested using this rationale to improve responses to cancer immunotherapies. In this review, we discuss the immune mechanisms operating in viral infections, show evidence that exogenous viruses and endogenous retroviruses in cancer may enhance tumor immunogenicity, dissect the epigenetic control of EREs, and point to interferon pathway activation in the tumor milieu as a promising molecular predictive marker and immunotherapy target. Finally, we briefly discuss current strategies to modulate these responses within tumor tissues, including the clinical use of innate immune receptor agonists and DNA demethylating agents.

## Introduction

Better understanding of the immune mechanisms operating in human cancers has led to breakthroughs in the way many cancers are treated with the introduction of effective immunotherapies, particularly those based on immune checkpoint blockade (ICB) (1). However, the therapeutic value of these approaches is still to be proven for many cancers, and even in cancer types showing high response rates to ICB, a significant proportion of patients may fail to respond to the treatment, highlighting the need of further investigation in order to enhance the treatment efficacy and broaden the application of this promising therapeutic approach (2).

Predictive biomarkers of immunotherapy response currently in use include the tumor expression of immune checkpoints, tumor mutational burden (TMB) and, to some extent, the density of tumor infiltrating lymphocytes (TILs) (3). While the expression of immune checkpoints indicates that ICB targets are active in the tumor milieu, TMB and TILs are correlates of tumor immunogenicity, reflecting the abundance of neoepitopes available for adaptive immunity recognition and the existence of active immune responses (3). Tumors with higher TMB and/or TILs, such as melanoma and lung cancers, the so-called “hot” tumors, are more likely to respond to immunotherapy (4). Conversely, current immunotherapies fail to elicit efficient antitumor immune responses in patients harboring “cold” tumors, including breast and prostate cancers (4, 5).

Despite this, these biomarkers do not reflect the tumor complexity and their clinical utility varies largely among different cancer types, making the search for global predictive biomarkers for ICB an attractive field (2). Also, the existence of cold tumors and the incapacity of ICB to elicit strong antitumor responses in this context highlight the need to investigate therapeutic strategies that may act as adjuvants to trigger or strengthen anti-tumoral immune response against poorly immunogenic cancers. Of note, the absence of strong innate immunity stimulators and the preponderance of autoantigens in tumor cells are factors associated with the inability to properly activate anti-tumor immune responses in these cancers (6).

Viruses are the most abundant and perhaps the most ancient biological entities in the world. These organisms need to infect cells to replicate and have evolved several mechanisms to store and propagate their genetic information. They are broadly divided into DNA and RNA viruses, the latter being further subdivided into retroviruses and single-stranded or double-stranded RNA viruses (6). Several viruses have the ability to infect human cells, and some of them can transform normal cells in multiple ways, such as through the induction of cellular proliferation and insertional mutagenesis, thus triggering cancer development and progression (7).

Among the major cancer-associated viruses are Epstein-Barr Virus (EBV), Human Papillomavirus (HPV), Hepatitis C virus (HCV), Hepatitis B virus (HBV) and Merkel cell polyomavirus (MCV) (7). Other viruses, such as Mouse Mammary Tumor Virus (MMTV) (8) and human cytomegalovirus (HCMV) (9), have debatable associations with human cancer development and could influence disease onset and progression, although no strong causal relationship has been shown.

Notwithstanding, some viruses and viral elements have been incorporated into the cellular genomes of different organisms over the course of evolution, probably due to infection in germinative cells. These reminiscent sequences, which make up about 40% of the human genome, are called endogenous retroviral elements (EREs), and their expression is tightly regulated by epigenetic mechanisms, to avoid the genomic instability they may cause through new insertional events (10, 11). Indeed, when this control is lost, EREs become aberrantly expressed and new insertional events lead to cancer initiation and progression by promoting genomic instability and driving evolution of transformed cells in multiple ways (12).

Interestingly, recent evidence suggests that tumor cells expressing viral elements or cancers aberrantly expressing EREs are more immunogenic and show increased TILs and cytotoxicity scores (13). Mechanistically, their replication intermediates are recognized by innate immunity sensors, enhancing the inflammatory response in the tumor microenvironment (TME), mainly through the induction of interferon (IFN) responses (13). Additionally, translated proteins from viruses and EREs might be presented *via* class I MHC on tumor cells and activate adaptive immune responses (14, 15).

Altogether, these data indicate that antiviral immune responses might boost antitumor immunity and may assist the discovery of biomarkers or therapeutic targets that can enhance immunotherapy response. Indeed, several therapeutic strategies aimed to induce a “viral mimicry” state in tumor cells are being investigated and have shown promising results in preclinical models and clinical trials, including DNA demethylating agents and agonists of innate immunity sensors (16, 17).

Thus, in this review, we discuss how viral elements or analogs can activate intra-tumoral immune responses mediated by IFNs and might serve as biomarkers and/or adjuvants for immunotherapies. We first summarize the immune response to viruses, discussing the activation of innate immune receptors that ultimately promotes IFN responses and activation of adaptive immunity; we next discuss the clinical meaning of interferon activation in tumors and the impact of cancer-associated viruses and EREs in the tumor immune microenvironment; finally, we present therapeutic approaches employing this knowledge to boost antitumor immune responses that are currently under development and clinical testing.

## Activation of IFN Signaling by Viruses

**Figure 1:**
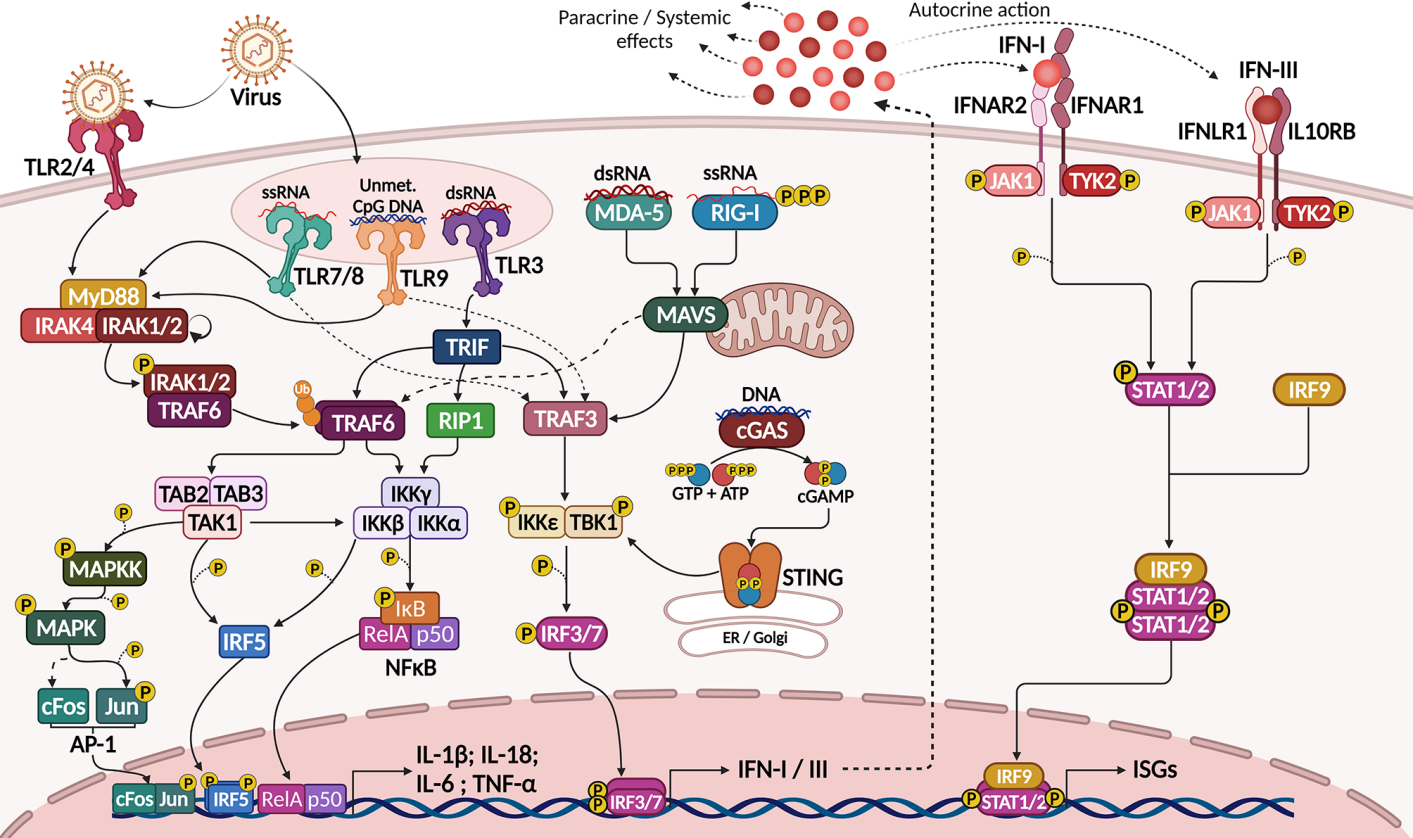
Activation of IFN-I/III response by viral sensing through PRRs. Sensing of viral molecules by plasma membrane (TRL4), endosomal (TLR3, 7, 8 and 9) or cytosolic (RLRs and cGAS) PRRs activate signaling pathways culminating in the expression of pro-inflammatory cytokines (IL-1β, IL-6, IL-18 and TNFα) by NF-κB, AP-1 and IRF5 transcription factors and of IFN-I and IFN-III by IRF3 and IRF7 (left). IFNs act though transmembrane receptors to activate STAT1, STAT2 and IRF9. This complex then translocates to the nucleus to govern the expression of several interferon-stimulated genes (right), which mediate an antiviral state leading to cell apoptosis, cytostasis, antigen presentation and expression of viral restricting factors. Created with BioRender.com.

Viruses elicit strong innate immune responses through activation of different families of pathogen recognition receptors (PRRs), including toll-like receptors (TLR), localized in plasma or endosomal membranes, and several cytoplasmic receptors that function as sensors for viral DNA (e.g. cGAS) and RNA (e.g. RIG-I and MDA-5). These receptors act through different, but convergent signaling cascades, culminating in the activation of transcription factors (TFs) that coordinate the expression of pro-inflammatory cytokines, including the expression of IL-1α, IL-1β, IL-18, IL-6 and TNFα *via* NF-κB activation and the expression of type I and type III IFNs, through the activation of interferon response factors (IRFs), mainly IRF3 and IRF7 (**Figure 1**) (18–21).

Type I IFNs (IFN-I) include the IFNα subfamily (which include 13 related proteins in humans), IFNβ, IFNϵ, IFNκ and IFNω, of which IFNα and IFNβ are the most prominent cytokines (22). Type III IFNs (IFN-III) include three closely-related IFN-λ, namely IFN-λ1, IFN-λ2 and IFN-λ3, previously known as IL-29, IL-28A and IL-28B, respectively (22, 23). Type I and type III interferons act through different pairs of class II cytokine receptors, triggering similar intracellular pathways that share the same final effector TFs to induce the so-called antiviral state within infected or susceptible cells (24). This mechanism prevents or restrains viral infection and replication through the induction of cytostasis and apoptosis, upregulation of the antigen-presenting machinery, expression of viral restriction factors, and recruitment and activation of immune cells (25).

Plasma membrane TLRs, such as TLR2 and TLR4, may respond to viral infections, since viral molecules, including surface proteins or glycoproteins, were shown to activate these receptors during viral adsorption and budding (18). However, antiviral innate immunity is commonly triggered when viral nucleic acids are recognized by endosomal TLRs, such as double stranded RNA (dsRNA) recognition by TLR3, single-stranded RNA (ssRNA) recognition by TLR7 and TLR8, or unmethylated CpG motifs by TLR9 (18, 19) (**Figure 1**).

Except for TLR3, all TLRs can recruit MyD88 when activated (TLR2 and TLR4 recruit the TIRAP adaptor protein before MyD88). MyD88 then interacts with IL-1R-associated kinase (IRAK)-1, -2 and -4, and phosphorylated IRAK1 interacts with and activates the ubiquitin ligase TNFR-associated factor 6 (TRAF6). TRAF6-mediated K63 polyubiquitination activates a series of kinases, including TGF-β-activated kinase 1 (TAK1) and IκB-kinase complex (IKK), which phosphorylate and mark IκB for degradation, thus releasing NF-κB for nuclear translocation. TAK1 and IKK also activate the Mitogen-activated protein kinase (MAPK) pathway and other TFs, such as Interferon Response Factor 5 (IRF5) and AP-1, which also interact with NF-κB (26). Altogether, these TFs coordinate the expression of pro-inflammatory cytokines such as IL-6, TNFα, IL-1β and IL-18, but not of IFNs (20) (**Figure 1**).

TLR3, otherwise, recruits TIR-domain containing adapter-inducing interferon-β (TRIF), which directly recruits and activates TRAF6 and the receptor-interacting protein 1 (RIP1), resulting in more potent NF-κB activation and pro-inflammatory cytokine production. TRIF also activates TRAF3, and this ubiquitin ligase is capable of activating TBK1 and IKKi kinases, which then activate IRF3 (20). Other TLRs, such as TLR7 and TLR9, also activate TRAF3 in response to viral nucleic acids in the endosome, but the main TF activated in this context is IRF7. Both IRF3 and IRF7 coordinate the expression of type I and type III interferons, being critical regulators of antiviral immune responses (18, 20) (**Figure 1**).

More recently it became clear that besides transmembrane TLRs, cytoplasmic proteins can act as virus sensors and elicit type I interferon responses. These include DNA sensors, such as cGAS, and RNA sensors, such as RIG-I, MDA-5 and LGP-2, collectively called RIG-like receptors (RLRs) (21). Also, AIM-2 and NLRP3 cytoplasmic proteins were shown to recognize intracellular DNA and dsRNA, respectively, being capable of activating the inflammasome complex to produce bioactive IL-1β and IL-18, further stimulating inflammation and the activation of immune cells (18). Several other proteins may act as sensors or accessory proteins in the recognition of viral nucleic acids in different contexts, but their role in antiviral immune responses is less clearly understood (18).

Different RNA sensors recognize RNA molecules differently depending on the RNA nature, and this also reflects how they recognize different viruses. RIG-I was shown to bind mainly 5’-triphosphate ssRNAs and short (up to 1kb) dsRNA, while MDA-5 recognizes long (>2 kb) dsRNAs preferentially (18). Both proteins have a C-terminal RNA-interacting domain, an RNA helicase domain, and two N-terminal caspase activation and recruitment domains (CARD), required for signaling. LGP-2 lacks the CARD domain, and a regulatory role was proposed for this protein; however, more recent evidence has shown that LGP-2 might potentiate RIG-I and MDA-5 responses to viral RNAs (27).

Upon RNA recognition, the CARD domains of RIG-I or MDA-5 are exposed, favoring the oligomerization of these proteins. Their CARD domain then interacts with the CARD domain from MAVS (also called IPS-1), a mitochondria-localized adaptor protein that forms aggregates and interacts with both TRAF3 and TRADD, thus recruiting TRAF6. TRAF3 then activates TBK1 and IKKϵ to induce IRF3/7 activity, while TRAF6 activates the IKK complex to promote NF-κB nuclear translocation and activity (18) (**Figure 1**).

Regarding recognition of cytoplasmic DNA, cGAS (an acronym for cyclic-di-GMP-AMP (cGAMP) synthase) is an enzyme that, when bound to DNA, synthesizes the second messenger cGAMP from GTP and ATP (28). This mediator then binds to STING, located in the endoplasmic reticulum (ER) membrane, and promotes its traffic to the Golgi complex where STING interacts with and activates the IKKϵ/TBK1 complex, also inducing the activation of IRF3/7 (**Figure 1**). Besides recognizing DNA from viruses, cGAS signaling can also be activated by RNA:cDNA hybrids generated during viral replication in the cytoplasm (29).

Collectively, IRF3 and IRF7 activation leads to the expression of type I and type III IFNs, which are secreted by infected cells and exert autocrine and paracrine signaling through the activation of transmembrane receptors: IFNAR1/IFNAR2 dimers for type I IFNs and IFNLR1/IL10RB dimers for type III IFNs. Both receptor dimers act through the JAK-STAT pathway, activating the receptor-associated kinases JAK1 and TYK2, which in turn activate STAT1, STAT2 and IRF9 (30). These TFs then translocate to the nucleus and regulate the expression of a plethora of genes, collectively known as interferon stimulated genes (ISGs), after binding to specific DNA sequences known as interferon-sensitive response elements (ISRE) (22) (**Figure 1**).

There are currently hundreds of well-defined ISGs and probably thousands of genes that are directly or indirectly regulated by IFNs (25). These genes include several viral restriction factors that act in different phases of viral replication, including fusion to the cellular and endocytic membrane, genome replication and protein translation, and are responsible to induce an antiviral cellular state, which is associated with enhanced proteasomal function, enhanced autophagy, cytostasis, apoptosis and improved antigen presentation through the upregulation of the antigen-presenting machinery in target cells (25).

Despite acting through similar molecular pathways and showing great overlap in the core genes they activate, IFN-I and IFN-III may differ in their physiological roles and spatiotemporal dynamics during antiviral responses. Overall, IFN-III responses act mainly in anatomical barriers, such as epithelial cells in mucosal surfaces that are in constant contact with pathogens, exerting their roles in a paracrine manner. IFN-III is deemed to evoke an initial, controlled, weaker and sustained response, whereas IFN-I emerges later and shows an acute and intense pattern of activation, stimulating more ISGs than IFN-III (30, 31). Factors explaining these differences include the restricted expression of IFNLR1 in epithelial cells, the ligand-receptor binding kinetics (32) and the differential activation of STAT-independent pathways in target cells (33), which are only beginning to be appreciated (30).

In addition to its effects on target cells, IFN signaling affects immune cells in multiple ways. IFN-I was shown to induce the production of chemokines (e.g., CXCL9, CXCL10 and CCL2), which enhance lymphocyte and macrophage recruitment, and cytokines (e.g., IL-15), that promote the maintenance of memory CD8^+^ T cells and natural killer (NK) cells (34). IFN-I also supports the differentiation of monocytes into dendritic cells (DC) and stimulates DC maturation by upregulating MHC-I and -II antigen-presenting machinery, costimulatory molecules, such as CD80 and CD86, and CCR7, a chemokine receptor that promotes DC migration to the T-cell zone of draining lymph nodes, where they can activate and polarize naive T cells into effector and/or memory T cells (34).

Upon antigen-driven activation, IFN-I has direct effects on T cells by favoring the polarization of naive CD4^+^ T cells towards the IFNγ-secreting Th1 phenotype and facilitating the activation, clonal expansion and IFNγ secretion of cytotoxic CD8^+^ T cells. NK cell activation and IFNγ secretion are also enhanced by IFN-I (34). In turn, IFNγ has a major role in coordinating immune responses by inducing the IgG production by B-lymphocyte-derived plasma cells, boosting macrophage function and enhancing DC antigen presentation (35).

On the other hand, IFN-I signaling in the absence of TCR signaling may lead to T cell apoptosis (36, 37), while chronification of IFN-I signaling triggers immunoregulatory mechanisms aimed to avoid severe tissue injury (38). This is achieved through the induction of immunosuppressive cytokines, such as IL-10 (39, 40), and exhaustion receptors and ligands on immune cells, such as PD-1 (41) and PD-L1 (42), among other suppressive molecules (43, 44), leading to a hyporesponsive state in activated T cells.

Similarly, IFN-III has direct effects on many immune cell populations, although these are not fully understood due to the non-ubiquitous expression of IFNLR1 on immune cells and the controversial data found in different models of immune response (30, 31). Notably, plasmacytoid and conventional DCs are able to both produce and respond to IFN-III, whereas other immune cells, such as neutrophils, macrophages and NK cells, are only capable to directly respond to it, but cannot produce IFN-III themselves (30, 31, 45).

An STAT-independent immunoregulatory role for IFN-III in neutrophils has been demonstrated in various contexts, including infectious and autoimmune diseases (30, 31, 45). DCs were shown to produce high levels of IL-12 in response to IFN-III (46). In NK cells, IFN-III was required for maximal IFN-γ production and antitumor activity (47), however it is not clear whether IFN-III has direct action on these cells (45). IFN-III has also shown to enhance IFN-γ production and reduce Th2 cytokines in CD4^+^ T cells, favoring a Th1 pattern (48, 49). In CD8^+^ T cells, IFN-III was shown to improve cell expansion and cytolytic activity (50).

However, controversial effects of IFN-III on T cells are shown depending on which inflammatory context has been evaluated: IFNLR1-deficient mice showed improved CD4^+^ and CD8^+^ T cell responses to transient acute LCMV infection, but showed diminished T cell responses and worse disease control in a chronic infectious scenario (51). Also, much of the IFN-III effects on T cells are attributed to its roles in the activation of the innate immunity compartment, especially DCs, and it remains to be determined the specific effects of IFN-III on T cells (45).

## Interferons in Tumor Immunity and Therapeutic Responses

**Figure 2:**
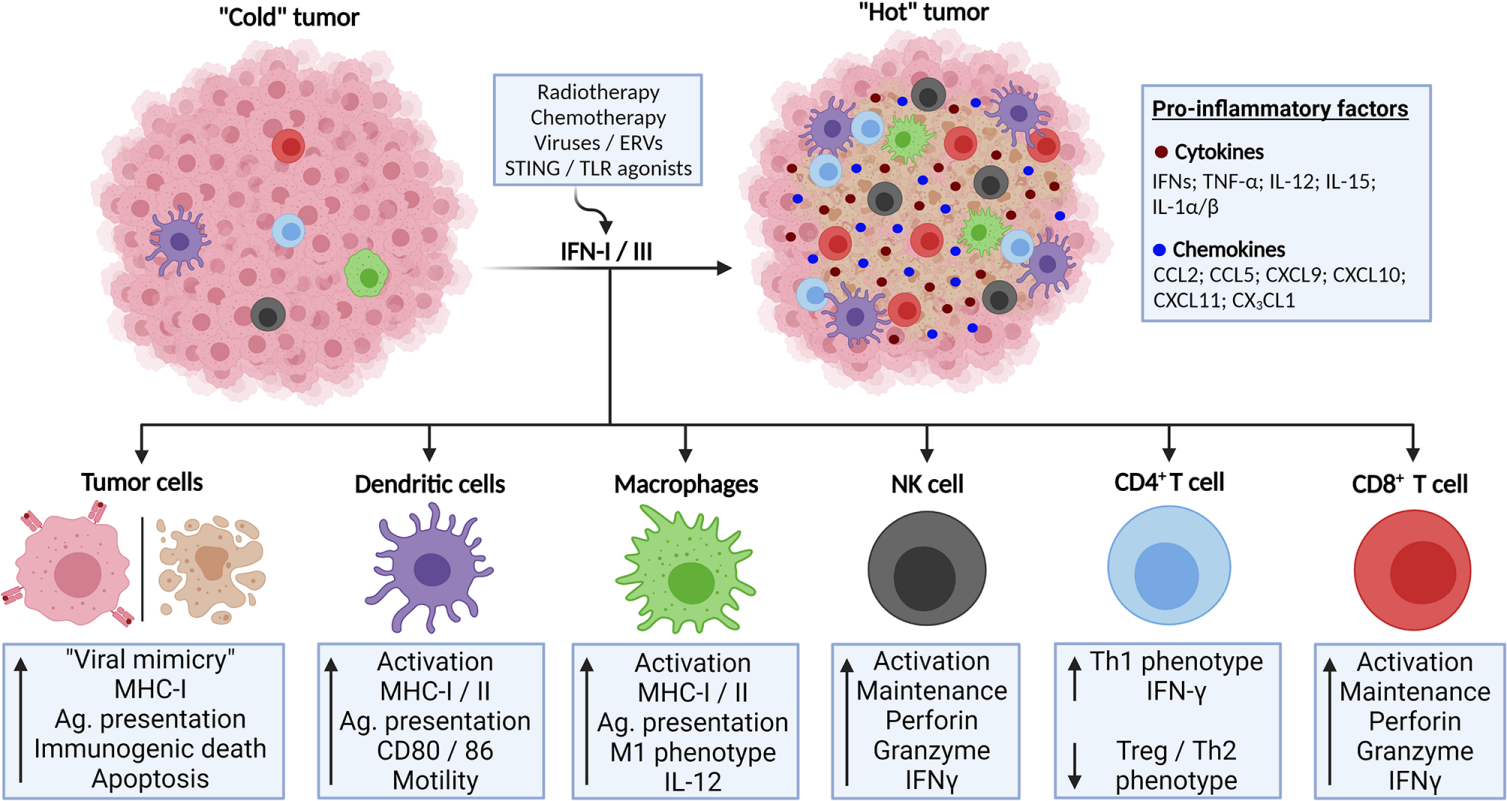
Activation of IFN-I/III responses in TME lead to enhanced anti-tumor response and tumor control. Poorly immunogenic (“cold”) tumors might be converted to highly infiltrated tumors (“hot”) through the activation of IFN-I/III responses. This might be accomplished through several strategies, such as genotoxic therapies and triggering of innate immunity receptors involved in antiviral responses. IFN-I/III mediate this phenomenon by its actions on tumor cells, inducing immunogenic cell death and enhanced antigen presentation as well as by its effects in activating anti-tumor immune cell populations, such as dendritic cells, T lymphocytes and natural killer (NK) cells. Created with BioRender.com.

Based on what has been discussed, IFN responses may exert anti-tumor effects through cell-intrinsic mechanisms, such as promotion of apoptosis, as well as the activation of immune mechanisms that optimally act against tumor cells, particularly those mediated by cytotoxic NK and T cells. In chronic scenarios, IFNs induce abundant expression of exhaustion markers on immune cells that can be targeted by ICB. Of note, many factors may activate IFN responses in the TME, including genotoxic therapies and engagement of PRRs by their natural ligands or agonists (**Figure 2**), which are currently being exploited as potential therapeutic agents, proving the crucial anticancer roles exerted by IFNs (52).

Early studies demonstrated that IFN-I administration improves the survival of tumor-harboring mice (53) and that endogenous IFN-I was required for tumor rejection (54, 55). Following the promising results observed in pre-clinical models, clinical trials with IFNα for human cancer treatment also showed efficacy in promoting regression of many tumor types (56), leading to IFNα2 approval as the first anticancer immunotherapy and its clinical use for many years to treat different cancers, including hematological malignancies (57, 58) and melanoma (59).

**Figure 3:**
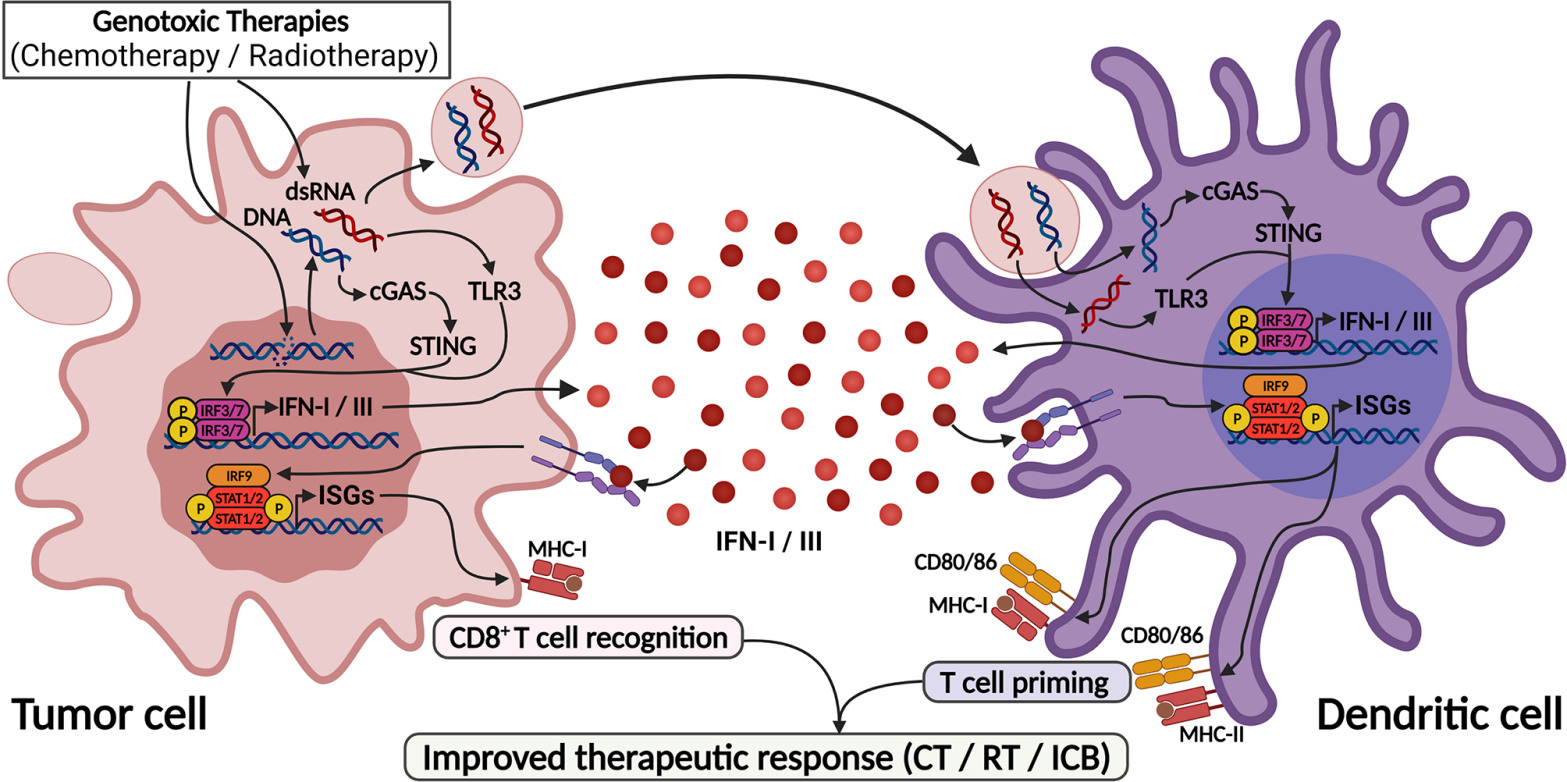
DNA-damaging agents trigger IFN-I/III responses through activation of cGAS/STING and TLR3 pathways. DNA-damaging agents cause DNA leakage into cytoplasm as well as induce dsRNA formation, leading to the activation of cGAS and TLR3. Also, blebs from dying cell containing DNA and dsRNA are captured by dendritic cells and also sensitizes cGAS and TLR3 in these cells. These pathways culminate in IFN-I/III production by both cells, leading to efficient dendritic cell activation and T cell priming, thus enhancing anti-tumor responses. This process mediates the response to genotoxic therapies and found the base for their use as adjuvants for ICB therapies. Created with BioRender.com.

IFN-I activation of immune responses has shown to be an essential trigger in mediating anti-tumor activities. IFN-I-induced transcripts correlated with T cell infiltration in human melanoma and mice models have shown that IFN-I can be produced by intratumoral DCs upon tumor implantation, being indispensable for the accumulation of intratumor cross-presenting dendritic cells and priming of CD8^+^ T cells (60) (**Figures 2**, **3**). Further data also suggested that IFN-I signaling has anti-metastatic properties by regulating epithelial-to-mesenchymal transition (EMT), angiogenesis, and the expression of cytokines, chemokines and their receptors in cancer (61).

The role of IFN-I in cancer immunoediting has also been elegantly demonstrated (62). Using mouse models for tumor transplantation and primary development, it was shown that non-immune-edited (immunogenic) sarcomas grown in *rag2*-deficient mice (which lack lymphocytes) were rejected by wild-type syngeneic mice; however IFNAR1 blockade abrogated this effect. Likewise, *ifnar1* knockout mice were more susceptible to carcinogen-induced tumor formation, and tumors grown in these animals were controlled when transplanted into WT animals, similarly to tumors generated in *rag2*-knockout mice. Additional experiments have shown that IFN-I signaling in immune cells, but not in tumor cells, is essential for tumor elimination (62). In conclusion, these data prove that IFN-I acting on immune cells is necessary for tumor immune responses, and the absence of this stimulus creates a permissive environment that promotes the growth and progression of non-immune-edited tumors (62).

Although the use of IFN for cancer treatment has decreased due to its related adverse effects and the advent of more effective targeted therapies, IFN-I exerts an important role in regulating cancer progression through the activation of antitumor immune responses and may serve as prognostic or therapeutic predictor biomarker in different cancers and therapeutic modalities (52). Relatedly, the efficacy of genotoxic therapies, including chemotherapy and radiotherapy, has been shown to largely rely on activation of the IFN-I pathway (63–67), while exogenous IFN-I stimulation could potentiate responses to these treatments (65, 68) (**Figures 2**, **3**). Of note, the unresponsiveness of IFN-I-deficient animals was attributed to their inability to activate dendritic and CD8^+^ T cells (52, 63, 66).

Accordingly, cGAS activation and IFN-I signaling in DCs are required for anti-tumor immune responses after radiotherapy (69) (**Figure 3**). Also, progression of tumor cells through cell cycling after the genotoxic stimulus led to the accumulation of cytoplasmic micronuclei that were recognized by cGAS, leading to IFN production and T cell priming, indicating that beyond DCs, tumor cell sensing of damaged DNA *via* STING pathway also contributes to IFN-I production and cancer immune responses (70) (**Figure 3**). Further, IFN-I activation was necessary to the abscopal (systemic) effects of radiotherapy in disseminated cancers, a phenomenon that is highly dependent on intratumor CD8^+^ T cell priming (70, 71).

In contrast, IFN-I pathway activation in tumor cells was associated with radiotherapy resistance, and *ifnar1*-deficient tumor cells were more susceptible to T cell killing in mouse models (72). Conversely, IFN-I signaling in tumor cells has been shown to upregulate indoleamine-2,3-dioxygenase 1 (IDO1) after radiation, which contributed to radiation resistance and inhibition of immune responses in tumors (73). These results highlight that (i) IFN-I activities and their potential impact on radiotherapy response varies according to the TME compartment (tumor *vs* immune cells) wherein this pathway is activated and (ii) IFN-I effects in boosting the radiotherapy response are immune-mediated.

In genotoxic chemotherapy with anthracyclines, TLR3 activation in tumor cells has been shown to largely contribute to IFN-I responses and tumor elimination after chemotherapy (66). Doxorubicin treatment induced an antiviral-like expression program, which was abrogated in *Tlr3*-knockout mice that did not respond to chemotherapy unless supplied with recombinant IFNα, IFNβ or CXCL10. Furthermore, an IFN-I-related expression signature predicted response to anthracyclines in multiple breast cancer cohorts (66). Importantly, DNA damaging agents might generate dsRNA, which can be recognized by TLR3 (74), and evidence has shown that ssRNA might also stimulate TLR3 through the formation of incomplete stem-loop structures (75), providing a rationale for TLR3 activation in this context (**Figure 3**). Additionally, IFN-I signaling was protective against breast cancer metastization, as it was required for NK and CD8^+^ anti-tumor functions in this scenario (76, 77). Accordingly, pre-chemotherapy IRF9 expression was consistently associated with treatment response and improved metastasis-free survival following chemotherapy in triple-negative breast cancer (78).

Since immunotherapies also rely on the efficient priming of intratumor immune responses, IFN-I signaling could also potentiate the efficiency of ICB (79). Indeed, STING-deficient mice were unable to respond to anti-PD-1 and anti-CTLA-4 in B16F10 melanoma model, and cGAMP administration along with ICB inhibited tumor and metastasis formation and delayed tumor growth (80). This process depends on IFN-I, and endothelial cells were shown as major contributors to this phenomenon by highly producing IFNβ1 in response to cGAMP (80). In the same melanoma model, cGAS-STING pathway and IFN-I production were necessary to potentiate anti-PD-L1 response, while STING- or cGAS-deficient mice were unable to respond to this therapy (81). Further, cGAMP administration increased DC and antigen-specific T-cell activation in a dose-dependent manner in WT and cGAS-deficient mice, but not in STING-deficient animals (81). Otherwise, the increased susceptibility of *ifnar1*-deficient tumor cells to T cell-mediated killing ultimately led to improved response to anti-PD-L1 ICB, once again highlighting the differing translational meaning of IFN-I activation in different tumor compartments (72).

Enhancement of ICB responses may be achieved by the use of genotoxic therapies before ICB, and several works have shown that the cGAS-STING-IFN-I pathway significantly contributes to this effect (70, 71, 82, 83). Cytoplasmic DNA accumulation following radiotherapy is necessary to properly activate the cGAS-STING pathway and potentiate ICB (70) (**Figure 3**). In line with this, it was recently shown that high radiation doses induce the exonuclease Trex1, which degrades cytoplasmic DNA and downmodulates cGAS-STING pathway, thus attenuating cancer immunogenicity; on the other hand, low dose radiotherapy (LDRT) effectively induces cytoplasmic DNA accumulation without Trex1 induction, and this efficiently stimulates IFNβ production and T-cell priming, enhancing the response to immunotherapy (83). Also, LDRT promoted immunologic infiltration in a murine lung cancer model characterized by the absence of T cell infiltration and induced cytotoxic CD4^+^ and CD8^+^ T cell populations (84). These findings were validated in immunotherapy-resistant human tumors, which after radiation were highly infiltrated by Th1 cell subpopulations (84).

Otherwise, recent reports have shown that prolonged IFN-I and II stimuli can upregulate and sustain the expression of multiple genes involved in T-cell exhaustion, including but not limited to PD-L1, thus mediating resistance to immunotherapies. Sustained IFN-I signaling and IFNβ expression were also associated with resistance to anti-PD-1 blockade therapy through the induction of NOS2 in immune and tumoral compartments of melanoma, promoting an accumulation of regulatory T cells and myeloid cells in tumor tissues (44). Conversely, in animals that do not respond to combinatorial treatment with anti-PD1 and anti-CTLA4, pharmacological inhibition of the IFN pathway could render immunotherapy-resistant tumors sensitive to either ICB monotherapy (43).

IFN-III functions in cancer are less clearly understood. The more restricted expression of IFNLR1 and IL10RB as opposed to IFNAR1 and IFNAR2 (which are ubiquitously expressed by nucleated cells) and the known attenuated inflammatory potential of IFN-III in comparison to IFN-I has led to the hypothesis that IFN-III administration would exert anti-tumor activities with less toxicity compared to IFN-I (30, 85). Indeed, IFN-III has shown *in vitro* and *in vivo* anti-tumor effects through the induction of tumor apoptosis and cell cycle arrest, inhibition of angiogenesis and activation of the immune compartment (86–90).

In a recent report, it has been shown that conventional dendritic cells 1 (cDC1) were responsible for type-III interferon production and that both intratumor cDC1 abundance and IFN-III expression were associated with improved prognosis in breast cancer patients (91). Further, IFN-III expression correlated with pro-inflammatory cytokines and chemokines in the tumor *milieu*, favoring Th1 polarization of intratumor immune responses (91). In agreement with this, *in vitro* stimulation of patient-derived cDC1 by TLR3 agonists led to the secretion of several inflammatory mediators, including IFN-β, IFN-γ, IL-12, CXCL10, CXCL11 and CX3CL1 (91). Also, IFN-III was required for optimal IFNγ production and antitumor activity of NK cells (47). Therefore, IFN-III may also exert direct and indirect anti-tumor activities and might hold potential as a biomarker and/or therapeutic target in cancer. However, the complex biology of IFN-III including its spatio-temporally restricted expression and its multifaceted roles on the immune system and human cancers, as well as its potential adverse effects must be further understood before its clinical use.

Altogether, these results emphasize the promising roles of IFN-I/III in cancer progression and control, as well as the complex nature of the dynamic IFN responses in the TME. Besides cytotoxic therapies, it became recently clear that other stimuli can also activate interferon responses, including infection by exogenous or oncogenic viruses and expression of EREs. In addition to the activation of innate immunity programs, viral sequences may generate proteins that can be recognized by the adaptive immunity, as discussed in the next sections.

## Exogenous Viruses as Triggers of Anti-Tumor Immune Responses

Over 50 years ago, Epstein et al. published their electron microscopy observations of EBV (Epstein-Barr virus) in cultured tumor cells of Burkitt’s lymphomas, which led to the discovery of the first human oncovirus (92, 93). Since then, much focus has been placed on discovering novel human oncoviruses, mainly using large-scale analyses of the complex genetic architecture of tumors (94, 95).

According to the last survey, up to 20% of all human cancers are related to oncogenic viruses, and some are recognized as human carcinogens by the International Agency for Research on Cancer (IARC) (96–98). Currently, at least eight well-characterized human oncoviruses are known: (i) Epstein-Barr Virus (EBV); (ii) human papillomavirus (HPV; types 16, 18, 31, 33, 35, 39, 45, 51, 52, 56, 58, and 59); (iii) Kaposi’s sarcoma-associated herpesvirus (KSHV), also known as human herpesvirus type 8 (HHV-8); (iv) human T-cell lymphotropic virus type 1 (HTLV-1); (v) Merkel cell polyomavirus (MCPyV); (vi) hepatitis B virus (HBV); (vii) hepatitis C virus (HCV); and (viii) human cytomegalovirus (HCMV) (97, 99–102). Of note, they exhibit a high genomic diversity, displaying either DNA (EBV, KSHV, HPV, HBV, and MCPyV) or RNA (HCV and HTLV-1) genomes. Interestingly, some viruses predominantly affect a particular gender (e.g., 90% of HPV-induced cancers occur in females) and have a broad tissue tropism.

Accumulating evidence indicates that the presence of oncoviruses is not sufficient to induce cancer development, as only a small proportion of infected individuals eventually develop cancer. Instead, it has been proposed they contribute to a multistep tumorigenesis and are implicated in many cancer hallmarks (95, 103–105), usually involving chronic inflammation (by generating reactive oxygen (ROS) and nitrogen (RNS) species), immunosuppression (e.g., co-opt cellular processes for replication and undermine immune recognition to support their propagation), sustained proliferative capacity through the expression of viral-encoded oncogenes by the infected cells (e.g., T antigen of MCPy; *LMP1, LMP2A, LMP2B, EBNA1, EBNA2* of EBV; *Tax* of HTLV-1; *E5, E6 and E7* of HPVs), genomic instability generated by insertional mutagenesis, control of the host epigenetic machinery (106), regulation of the mitochondrial function of infected cells (107) and alteration of tumor suppressor (e.g., p53 and pRB pathways) and host signaling (e.g., PI3K–AKT–mTOR; MAPK; Notch; WNT/β-catenin; NF-κB) pathways (7, 108, 109).

Alternatively, the “Hit-and-Run” mechanism proposes that oncoviruses may act as initial triggers for cancer development (the “hit”), and the viral genome subsequently disappears (the “run”) after accumulation of new mutations in the host cell throughout carcinogenesis (110). However, clear experimental evidence is still required to support this hypothesis, although this phenomenon has been observed in an animal model (111). Interestingly, the presence of a mutational pattern consistent with off-target activity of the IFN-induced antiviral APOBEC3 cytidine deaminases in multiple human cancers (112) might constitute a link among viral infections, antiviral immunity and carcinogenesis that may operate through the hit-and-run mechanisms (113, 114). Indeed, association amongst APOBEC3 expression, mutational signature and viral infection has been established for cancers associated with HPV (115–117), polyomaviruses (118) and EBV (119). Additionally, recent work has given support to the idea that the genomic integration of some oncoviruses may be associated with increased somatic copy-number alterations and mutations (such as *TP53, CDKN2A* and *TERT* mutations in head and neck cancers) nearby the integration sites, and higher abundance of T cell and M1 macrophage populations in head and neck tumors (95), providing new insights into the causality of oncoviruses.

The TME plays a crucial role in modulating immune responses during cancer development, thereby influencing therapeutic outcomes and patient prognosis. In this context, several lines of evidence support that some oncoviruses that have been integrated into the genome of tumor cells might also directly or indirectly impact the intratumoral infiltration of immune cells and antitumor immunity. **Table 1** summarizes the findings of studies investigating the influence of oncoviruses on intratumor immune infiltrates. Overall, virus-associated cancers had increased infiltration of immune cells, particularly CD8^+^ T cells, and the presence of oncoviruses is commonly associated with better prognosis. Also, in some cases, notably for HCC and GC, viral infections were associated with high levels of regulatory T cells (**Table 1**). Notwithstanding, some cancers associated with viruses, such as HPV-associated HNSCC, have increased lymphoid aggregates composed of B and T cells known as tertiary lymphoid structures (127, 129), which may serve as niches for antigen presentation in the TME and are associated with improved prognosis and response to ICB in multiple cancers (145).

**Table 1:** Immune and clinical findings of virus positive versus virus negative tumors among different cancer types.

Virus^a^	Cancer^b^	Positive samples	Material^c^	Method^d^	Immune cell infiltrate*	Clinical outcome*	Reference
**MCPyV**	**MCC**	34/49 (69.38%)	FFPE	IHC	↑ CD4+ and CD8+ T cells	↑ OS	(120)
					↑ PD-L1 expression		
	**MCC**	85/116 (73.3%)	FFPE	IHC	↑ CD3+, CD8+, CD16+, FoxP3+, and CD68+ cells	↑ OS	(121)
	**MCC**	85/132 (64%)	FFPE	IHC	↑ CD8+ cells	↑ OS and PFS	(122)
					↑ PD-L1 Expression		
	**MCC**	84/134 (62.68%)	FFPE	IHC	↑ CD8+ and FOXP3+ cells	–	(123)
	**MCC**	38/49 (79.2%)	FFPE	IHC	No association	↑ OS (high CD8+ T cell infiltrate)	(124)
						↓ OS (high viral load)	
**HPV**	**HNSCC**	13/27 (48%)	FFPE	IHC	↑ CD3+, CD4+, CD8+, CD20+, and PD-1+ cells	–	(125)
	**HNSCC**	30/502 (5.97%)	RNA	RNA-seq	↑ activated NK cells, Monocytes cells, Macrophages M0 cells, resting Dendritic cells, Neutrophil cells	–	(126)
					↑ CXCL9 expression		
	**HNSCC**	9/63 (14.28%)	PBMC	FACS scRNA-seq	↑ CD4+ TFH, Germinal center (GC) B cells	↑ PFS (GC B cells)	(127)
	**HNSCC**	11/38 (28.9%)	PBMC	FACS	↑ CD45+ lymphocytes and B cells (CD19+/CD20+)	–	(128)
				IHC	↓ CD86+/CD21− antigen-presenting B cells		
	**OPSCC**	63/72 (87.5%)	FFPE	FACS	↑ CD20+ B cells	–	(129)
			Fresh tissue	IHC	↑ CD8+ T cells		
	**SCC**	8/31 (26%)	FFPE	IHC	↑ CD8+	–	(130)
	**HNSCC**	8/34 (24%)	PBMC	FACS	↑ CD45+ lymphocytes	–	(131)
			FFPE	IHC	↑ PD-1+ T		
**EBV**	**GC**	32/571 (5%)	FFPE	IHC	↑ CD8+ and FOXP3+ T cells	↑ OS	(132)
	**GC**	12/71 (17%)	Fresh tissue	RNA-seq (TCGA)	↑ CD8+ and NK cells	–	(133)
			FFPE	qRT-PCR	↑ ISGs		
				IHC			
	**GC**	–	FFPE	IHC	↑ Proliferating (Ki67+) CD8+ T cells	–	(134)
	**GC**	45/90 (50%)	Fresh tissue	IHC	↑ Tregs	–	(135)
			FFPE	FACS	↑ CCL22 expression		
	**GC**	6/43 (14%)	FFPE	IHC	↑ CD8+ and CD4+ cells and macrophages	–	(136)
	GC	28/129 (22%)	FFPE	IHC	↓ M2 macrophages (CD204+ cells)	↑ OS	(137)
	GC	20/48 (42%)	FFPE	IHC	↑ GzB7+CD8+ T cells	↓ LN metastasis	(138)
					↑ MHC-II		
**HBV**	**HCC**	24/46 (52.17%)	Fresh tissue	scRNA-seq	↑ Trm (PD-1-low/TOX-low)	↑ RFS	(139)
			PBMC				
	**HCC**	361/411 (88%)	FFPE	IHC	↑ CD8+	–	(140)
					↑ PD-L1 TIL		
	**HCC**	123/123 (100%)	PBMC	FACS	↑ Treg (CD4+CD25+FoxP3+) TILs	↓ OS associated with Tregs	(141)
				IHC	↓ CD8+ TILs		
					↓ Perforin, granzyme A and B in CD8^+^ T cells		
	**HCC**	12/20 (60%)	Fresh tissue	IHC	↑ PD-L1	–	(142)
**HCMV**	**GC**	504/573 (88%)	FFPE	IHC	↑ CD4+, CD8+, CD66b+, and CD163+ cells	↑ OS	(143)
				TMA		↑ RFS	
				RNA-seq			
	**CRC**	43/95 (45.3%)	Fresh tissue	PCR array	↑ Th17 signature	↓ DFS	(144)

*Unless otherwise specified, comparisons refer to virus-positive versus virus-negative tumors.

a MCPyV, Merkel-cell polyomavirus; HPV, Human papillomavirus; EBV, Epstein-Barr virus; HBV, Hepatitis B virus; HCMV, Human cytomegalovirus.

b MCC, Merkel cell carcinoma; HNSCC, Head and neck squamous cell carcinoma; OPSCC, Oropharyngeal squamous cell carcinoma; SCC, Squamous cell carcinoma; GC, gastric cancer; HCC, Hepatocellular carcinoma; CRC, Colorectal cancer.

c FFPE, Formalin-fixed paraffin-embedded tissue; RNA, Ribonucleic acid; PBMC, Peripheral blood mononuclear cells.

d IHC, Immunohistochemistry; RNA-seq, RNA sequencing; FACS, Fluorescent activated cell sorting (flow cytometry); qRT-PCR, quantitative reverse transcriptase polymerase chain reaction; TMA, tissue microarray; PCR, Polymerase chain reaction.

Taking advantage of The Cancer Genome Atlas (TCGA) data (146), Rooney et al. have shown that EBV infection in stomach cancer and HPV infection in head and neck squamous cell carcinoma (HNSCC) were associated with high cytolytic scores, which were indicative of improved cytotoxic T cell activity (13). Subsequent analysis also suggested that, apart from HBV-infected liver hepatocellular carcinomas (LIHCs), virus-infected tumors had higher cytolytic cell infiltration when compared to non-infected tumors of the same type (147). Also, decreased TCR clonality was observed in EBV-infected stomach cancers, suggesting that antigen-driven clonal expansion of T lymphocytes was taking place in these tumors (147).

T cell co-stimulatory and inhibitory receptors and ligands, as well as memory and resident T cell markers, were increased in tumors positive for HPV, EBV and CMV, but not HBV^+^ LIHC (148). Of note, gene expression signatures associated with viral infection were predictive of better overall survival in HNSCC and bladder cancer (147), and high T cell infiltration was predictive of overall survival in HPV^+^ HNSCC but not in HPV^-^ samples (148). In gastric cancer, EBV^+^ tumors also had increased expression of immune checkpoints (149) and IRF3 gene signature (150). Using single-cell transcriptomics, it has recently been shown that malignant cells from EBV^+^ gastric cancers were distinguished by a gene expression signature composed of IFN-I-activated and antigen-presenting molecules (151).

Oncovirus-specific T cells and epitopes have also been characterized in virus-associated tumors, such as MCPyV-associated Merkel cell carcinoma (152); HPV-associated cervical cancer (153, 154) and HNSCC (155–158); EBV-associated nasopharyngeal carcinoma (159, 160) and gastric cancer (161); and HBV-associated hepatocellular carcinomas (139, 162). Surgical removal of HPV-positive tumors followed by HPV vaccination has shown promising results to prevent cervical cancer recurrence (163, 164) and the use of virus-specific T cells or T cells engineered to recognize virus-derived antigens have shown promising results in preclinical models, case reports and clinical trials in multiple cancers (165–167), suggesting that recognition of viral antigens by cells of the adaptive immune system is a crucial feature controlling the growth and therapeutic response of virus-associated cancers.

However, the boosting of anti-tumor immune responses by viruses is not restricted to oncogenic viruses: recent reports highlight that commensal viruses are protective against tumors. In murine models of cutaneous squamous cell carcinoma (cSCC), infection by mouse papillomavirus 1 (MmuVP1) prior to carcinogen-induced skin cancer development delayed tumor formation and progression and prolonged survival in a CD8^+^ T cell-dependent fashion (168). Conversely, higher β-HPV RNA and DNA were found in cSCC of immunosuppressed patients relative to their immunocompetent counterparts, and CD8^+^ T cells isolated from human tumor tissues responded to peptides from the E7 protein of commensal β-HPV, but not to the oncogenic HPV-16, suggesting that immunocompetent patients had active immune responses against β-HPV operating in the TME (168).

Of note, virus-specific bystander T cells are abundantly found in the tumor core of murine models of cancer (169) and human tumors (170, 171). These cells constitute a tissue-resident memory T cell population expanded by multiple viral infections throughout host lifetime that may be recruited to tumor tissues through CXCR3 interaction with intratumorally-secreted CXCL9 and CXCL10 (172) and can be (re)activated by IFN-I stimulation (173). Recent reports indicated that bystander T cells are less prone to terminal exhaustion than tumor-antigen-specific T cells (169), and their activation in the TME may boost antitumor immunity not only through secretion of pro-inflammatory factors, but also through antigen-independent killing of tumor cells in a NKG2D-dependent manner (174, 175).

Notably, repeated exposure to cognate viral-antigen makes CD8^+^ T cells more sensitive to antigen-independent bystander activation in cancer, suggesting that CD8^+^ T cells responsive to common human viruses, such as CMV, EBV and influenza, may be the best targets to boost antitumor immunity (176). Consistently, lung infection by influenza virus protected mice against lung colonization of B16F10 melanoma cells injected intravenously, and hospitalization for influenza infection during cancer treatment was protective against death in lung cancer patients (177). In this context, pre-clinical models have also shown that intratumoral administration of non-adjuvanted influenza vaccine delayed tumor growth and boosted the response to anti-PD-1 immunotherapy, mainly by recruiting and activating cross-presenting dendritic cells and CD8^+^ T cells (177). In another murine model, non-adjuvanted vaccination resulted in reactivation of bystander virus-specific tissue-resident memory T cells as well as dendritic and NK cells, which favored tumor growth arrest, synergizing with ICB (171). Importantly, at distant anatomical sites from tumors, viruses can promote tumor growth by shunting tumor-reactive immune cells to the infected tissue (178).

Collectively, these data show that virus-infected tumors have increased immunogenicity and that localized (but not distal) activation of virus-specific T cells might restrain tumor growth by directly killing tumor cells, as known for oncoviruses. In addition, activation of intratumor immune responses by bystander virus-specific tissue-resident T cells may boost anti-tumor immunity.

## Endogenous Retroviral Elements and Tumor Immunity

Endogenous retroviral elements (EREs) are retrovirus-derived DNA sequences incorporated into the genome of host species through ancient infection of germinative cells. These elements comprise approximately 45% of the entire human genome and can be broadly classified into long terminal repeat (LTR) and non-LTR EREs (10). LTR-EREs can be further classified into (i) endogenous retroviruses (ERVs, or HERVs, in the case of human ERVs), in which LTRs flank complete or partial classical proviral sequences; (ii) mammalian-apparent LTR transposons (MaLRs); and (iii) solo LTRs. Non-LTR sequences otherwise comprise long and short interspaced elements (LINEs and SINEs, respectively) and *Alu* elements (10).

These elements are unable to generate provirus structures due to mutations in their ORFs actively generated through cellular viral restriction enzymes and therefore they cannot infect other cells. However, their ORFs can be transcribed into RNA and translated into proteins, and many of them hold the capacity to generate copies of themselves and reintegrate into the cellular genome, being called transposable elements (TEs) or transposons. Type-I TEs replicate into RNA sequences and use reverse transcriptase to copy themselves into DNA prior to reintegration, while type-II TEs simply encode enzymes called transposases to be excised and reintegrated into the genome as DNA (12).

Given the random nature of insertion events, EREs can modify the cellular genomic architecture and trigger many phenotypic alterations: their insertion into coding regions can inactivate genes, while intergenic re-insertions can modify the expression of proximal genes, since EREs can act as cryptic promoters. Also, the sequence similarity shared between EREs, especially between ERVs, in different chromosomal *loci* can promote non-homologous recombination, resulting in chromosomal aberrations such as translocations, deletions and inversions (179, 180).

In addition to the carcinogenic role on genomic instability, EREs may trigger immune responses when aberrantly expressed through the activation of innate immune receptors (179, 181), and their expression is associated with increased immune infiltrates and immune gene signatures in multiple cancers (13, 14, 182). Similarly, the enhanced immunogenicity of breast cancer models after treatment with CDK4/6 inhibitors (183) and the natural immunogenicity of rhabdoid tumors (184) were attributed to the expression of EREs and activation of IFN-I and III signaling pathways.

Due to the potential in generating genomic instability, EREs are tightly controlled by epigenetic mechanisms (185–187). Regarding ERVs, it has been shown that young sequences (i.e., those that have been incorporated into the genome more recently in the evolutionary history and predominantly contain more conserved ORFs) are repressed mainly by methylation of CpG islands in their LTRs and could have their expression reactivated by DNA hypomethylating agents (DHA) such as 5-aza-2′-deoxycytidine, while older ERVs are less enriched in CpG islands and are mainly controlled by histone modifications, particularly histone H3 lysine 9 trimethylation (H3K9me3) (188). Interestingly, the natural resistance to carcinogenesis observed in blind mole rats has been recently attributed to their loose epigenetic control of EREs: since their tissues express low levels of DNA methyltransferase 1 (DNMT1), uncontrolled proliferation leads to loss in control of EREs expression leading to cGAS-STING triggering, IFN-I response activation and cell death (189).

Strikingly, treatment of tumor cells with DHA and histone deacetylase inhibitors (HDACi) led to the expression of EREs that could be detected through MDA-5 in the form of cytoplasmic dsRNA, formed by hybridization between complementary RNA strands expressed from bidirectional transcription of EREs (190), homologous EREs transcribed from different *loci* and from loop structures formed by transcription of DNA stretches containing homologous antisense-oriented EREs sequences (187). This ERE detection by cytoplasmic sensors induces an IFN-mediated antiviral state marked by enhanced cancer cell apoptosis and activation of innate and adaptive immunity that could favor responses to ICB with anti-CTLA-4 (191–193). Further research identified *Alu* elements as major source of dsRNA activating MDA-5, whereas ADAR1, an interferon-induced adenosine deaminase that takes part in innate immunity to viruses, has been thought to be a negative regulator of these elements, thus downmodulating MDA-5 and IFN responses (194). Notably, ADAR1 depletion in patient-derived colorectal cancer cells increased their *in vitro* and *in vivo* sensitivity to DNA hypomethylating agents (194).

ERVs have also been shown to significantly contribute to the pool of tumor antigens recognized by CD8^+^ T cells, therefore also contributing to the activation of adaptive anti-tumor immune responses. In renal cell carcinoma (RCC), HERV-E is selectively expressed in cancer cells lacking the VHL tumor suppressor (195), and peptides derived from its envelope protein have been shown to bind HLA-A*0201 and stimulate patient-derived CD8^+^ T cells (196). Interestingly, following hematopoietic stem cell transplantation, tumor-directed immune responses consistent with graft versus tumor reactions targeted HERV-E epitopes and promoted regression of metastatic cancer in RCC patients (197). In clear cell RCC (ccRCC), immunogenic HERVs were associated with increased expression of immune signatures and response to anti-PD-1 and anti-PD-L1 (198). Notably, a recent report identified and validated 30 epitopes derived from HERV 4700 that could be recognized by CD8^+^ T cells from ccRCC patients and whose expression was also associated with improved response to anti-PD-1 therapy (182).

Indeed, using murine cell lines and tumor models, it has been shown that intergenic regions mapping transposable elements were the main source of epitopes targeted in anti-tumor immune responses boosted by immunotherapy, which could also be efficiently used in vaccination protocols, improving survival (199). Accordingly, transcripts overlapping LTR sequences in human cancers were thoroughly characterized in the TCGA database, wherein LTR-overlapping transcripts with prognostic implications have been identified, particularly in melanoma. Immunopeptidome of melanoma biopsies revealed some LTR-overlapping products capable of binding to MHC-I alleles (200). Of note, recognition of a HERV-derived epitope by CD8^+^ T cells in a melanoma patient had also been previously demonstrated (201).

In another report, EREs transcript expression in TCGA pan-cancer datasets was also associated with increased expression of immune-related genes, and treatment of glioblastoma cells with DHA promoted increased presentation of ERE-derived peptides *via* MHC-I (14). HERV-derived epitopes have also been characterized in both ovarian cancer (202) and hematological malignancies from the myeloid lineage (203). Multi-dimensional proteogenomic studies integrating data from exome sequencing, whole bulk and single-cell transcriptomics, sequencing of ribosome-bound RNA (Ribo-seq) and mass spectrometry-based immunopeptidome confirmed EREs as important epitope sources in cancers (204).

In summary, these data indicate that modulation of EREs expression in tumor cells by epigenetic modulators, such as DHA and HDACi, holds promising effects in anti-cancer therapies, especially ICB, since it can enhance tumor-cell death and immunogenicity by the activation of IFN-I and -III responses in the TME or unleashing tumor-associated antigens that could be targeted by cytotoxic T cells activated in this therapeutic modality (204–207).

## Antiviral Immune Responses and Cancer: Therapeutic Opportunities

Knowledge regarding the anti-tumor roles of antiviral immunity through the activation of IFN-I and IFN-III has opened new opportunities for cancer therapeutics. Currently, there exists an abundance of anti-cancer agents aimed at modulating these responses in TME under investigation in preclinical models and clinical trials, either as single agents or adjuvants for other immunotherapy (**Table 2**).

**Table 2:** Recruiting studies testing immunotherapy in combination with drugs that induce antiviral-like responses.

Drug/Product	Trial	Phase	Indication^a^	Combination	Identifier
**Oncolytic viruses**					
ADV/HSV-tk	STOMP	II	TNBC	Pembrolizumab (anti-PD1)	NCT03004183
			NSCLC	SBRT	
Pelareorep	IRENE	II	TNBC	Retifanlimab (anti-PD1)	NCT04445844
Pexastimogene Devacirepvec (Pexa-Vec)	ISI-JX	I	Solid tumors	Ipilimumab (anti-CTLA4)	NCT02977156
Ad/MG1-E6E7	Kingfisher	I	HPV related tumors	Atezolizumab (anti-PD-L1)	NCT03618953
TBio-6517	RAPTOR	II	Solid tumors TNBC MS-colorectal cancer	Pembrolizumab	NCT04301011
Pelareorep	BRACELET-1	II	Metastatic HR+/HER2- breast cancer	Avelumab (anti-PD-L1)	NCT04215146
				Paclitaxel	
RP-1	CERPASS	II	Squamous skin cancer	Cemiplimab (anti-PD1)	NCT04050436
OBP-301 (Telomelysin)		II	HNSCC	Pembrolizumab	NCT04685499
Pexastimogene Devacirepvec (Pexa-Vec)		II	Renal cell carcinoma	Cemiplimab	NCT03294083
**Interferon**					
Recombinant interferon alpha 2b-like protein		II	Fibrolamellar hepatocelularcarcinoma	Nivolumab (anti-PD1) 5-fluorouracyl	NCT04380545
**STING agonists**					
TAK-676		I	Solid tumors	Pembrolizumab	NCT04420884
E7766	INSTAL-101	I	Lymphoma		NCT04144140
			Solid tumors		
SNX281		I	Lymphoma	Pembrolizumab	NCT04609579
			Solid tumors		
**Epigenetic modifiers**					
Vorinostat	PEVOsq	II	Squamous cell carcinoma (lung, HN, vulva, penis, anus, cervix)	Pembrolizumab	NCT04357873
Entinostat	MORPHEUS HR+BC	II randomized (multiple arms)*	Breast cancer HR+/HER2-	Atezolizumab	NCT03280563
Epacadostat	POD1UM-204	I/II	Endometrial cancer	Retifanlimab	NCT04463771
Epacadostat		II	HNSCC	Pembrolizumab	NCT03823131
				Electroporation	
Tinostamustine	ENIgMA	I	Pancreatic cancer	Nivolumab	NCT03903458
Decitabine		II	Breast cancer HER2-	Pembrolizumab	NCT02957968
Tazemetostat		I/II	Urothelial carcinoma	Pembrolizumab	NCT03854474
**TLR9 agonists**					
SD-101		I	Non-Hodgkin’s lymphoma	BMS986178 (anti-OX40)	NCT03410901
CMP-001		II (randomized)	Melanoma	Nivolumab	NCT04401995
**TLR3 agonist**					
Poly-ICLC		I/II	Colorectal cancer	Pembrolizumab	NCT02834052
Poly-ICLC		I	Prostate cancer (neoadjuvant)		NCT03262103

*The combination of entinostat and pembrolizumab is one of the arms in this trial.

a TNBC, triple-negative breast cancer; NSCLC, non-small cell lung cancer; HPV, human papillomavirus; MS, microsatellite-stable; HNSCC, head and neck squamous cell carcinoma; HR, hormone receptor; HER2, human epidermal growth receptor 2; HN, head and neck.

This is not intended to be an exhaustive list of all ongoing clinical trials that explore antiviral response to modulate or elicit anti-tumoral immune response. For a more comprehensive list of trials, please access https://clinicaltrials.gov.

Also, the capacity of some viruses to directly induce cancer cell death and tumor regression, the so-called oncolytic viruses, has been largely reported and various families of viruses, including adenovirus, reovirus, Seneca Valley virus and herpesvirus, have been largely exploited therapeutically (208–211). Currently, Zika virus (ZIKV), a neurotropic flavivirus that promotes death of embryonal neural cells and recently caused a microcephaly epidemic in South America (212), is being investigated for the treatment of embryonal central nervous system tumors, showing great oncolytic activity in preclinical models (213–215). Of note, at least part of this effect is mediated by activation of CD8^+^ T cells and this treatment may synergize with ICB (216). Nevertheless, no single unmodified virus has shown consistent clinical activity to merit approval.

The only oncolytic virus that has received approval from some regulatory agencies is talimogene laherparepvec (T-VEC) for the treatment of unresectable advanced cutaneous melanoma (217). T-VEC is an engineered herpes simplex virus (HSV) type I designed to selectively replicate within and lyse tumor cells and concomitantly induce regional and systemic antitumor immunity. Two viral genes are deleted to attenuate neurovirulence, enhance tumor replication selectivity and improve antigen presentation of viral proteins in infected cells. T-VEC also carries the GM-CSF gene to improve the recruitment and activation of antigen-presenting cells and facilitate the adaptive T cell response. The intralesional administration of T-VEC was compared with GM-CSF administered subcutaneously in a randomized phase III trial that recruited stage IIB and IV melanoma patients. T-VEC was associated with better durable response rates (16.3% x 2.1%, p<0.001) and longer median OS (23.3 x 18.9 months, p=0.051) (217). Other modified viruses are under investigation for many different indications, as shown in **Table 2**.

As highlighted, interferon is produced by cells in response to viral infections and induces many modifications that restrain virus proliferation and contribute to their elimination. Similarly, interferon-dependent modifications are associated with tumor growth control and induction of effective antitumor immune response. Following this rationale, interferon has been used in the past to treat many different cancers, including renal cell carcinoma (218–220), melanoma (221–224), lymphoma (225), chronic myelogenous leukemia (226) and multiple myeloma (227), and although antitumor activity has been demonstrated, only a few patients benefit from such treatment. Indeed, some patients derived long-term disease control, albeit at the expense of significant systemic toxic effects, resulting in symptoms such as fever, chills, fatigue, depression and anorexia (52), therefore limiting its widespread use in clinical practice, mainly after the clinical implementation of ICB-based therapies.

Since most tumors present high genomic instability and defects in DNA repair genes, they usually exhibit a great amount of cytoplasmic DNA capable of triggering cGAS-STING pathway activation and interferon production. STING agonists have been tested to treat cancer. DMXAA (ASA-404) or valdimesan was the first STING agonist tested. At that time, DMXAA was used at high doses as a vascular disrupting agent in mouse models, and regardless of promoting vascular necrosis and tumor regression, the TME remained poorly immunogenic, since high doses of DMXAA induced T cell apoptosis, a phenotype that favors rapid tumor regrowth (16). Despite this, a phase III trial compared carboplatin and paclitaxel with or without ASA-404 in metastatic NSCLC. The addition of ASA-404 did not improve overall survival, progression-free survival, or overall response rate (228). It was subsequently shown that DMXAA does not bind properly to human STING (229). Although this class of drugs has shown great potential, there are no STING agonists approved yet. A great limitation of the STING agonists currently available is that they must be administered intratumorally; nevertheless, new drugs that can be given systemically are under investigation (**Table 2**) (16). Similarly, agonists of other innate immune sensors (TLR9 and TLR3 agonists) are also being tested in clinical trials (230) (**Table 2**).

**Figure 4:**
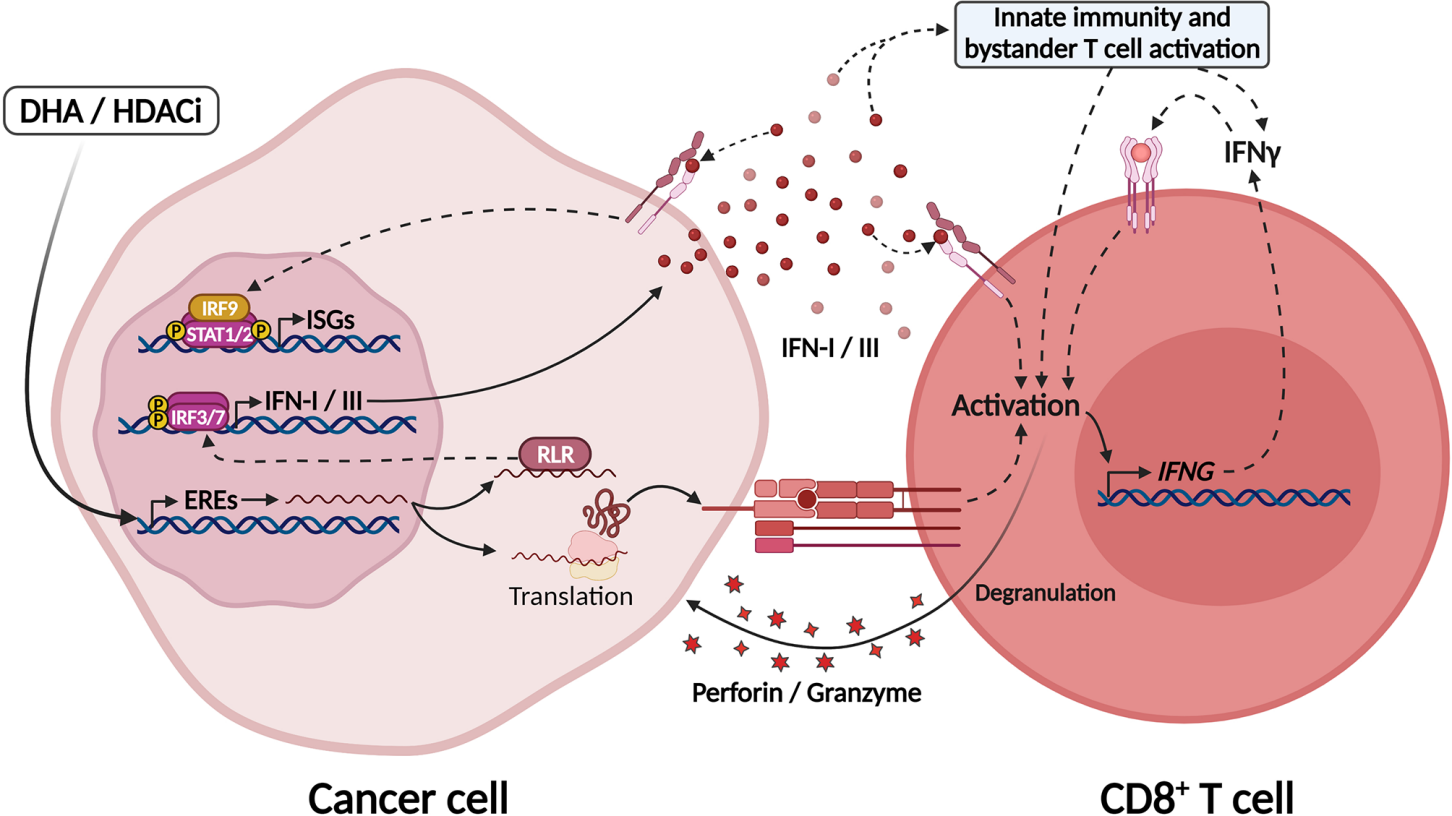
Epigenetic modulators unleash EREs expression culminating in IFN-I/III secretion through activation of MDA5. Treatment with DNA hypomethylating agents (DHA) and histone deacetylase inhibitors (HDACi) lead to chromatin modifications and unleashing of endogenous retroviral elements (EREs) in tumor cells. The intermediates formed by these elements during their replication sensitize RIG-like receptors in the cytosol, such as MDA-5 and RIG-I, which activate IRF7/9 and the production of IFN-I/III. ERE-derived RNAs might also be translated in tumor cells generating tumor-associated antigens that may be recognized by CD8+ T cells. In this manner, DHA and HDACi increase tumor immunogenicity and might be explored as adjuvants for ICB therapies. Created with BioRender.com.

HDACis and DHAs, such as DNA methyl-transferase inhibitors, are epigenetic modifiers that can promote alterations in both tumor cells and their microenvironment and ultimately enhance ICB efficacy. The synergistic effect of these drugs and ICB can be partly explained by the re-expression of EREs (“viral mimicry”) in tumor cells and the induction of interferon responses (191, 206, 231) (**Figure 4**). Furthermore, these epigenetic modulators can exert direct effects on immune cells: HDACi and DHA treatment were shown to selectively deplete myeloid derived suppressor cells (MDSCs), leading to improved response to ICB in preclinical models (232); also, DHA treatment directly enhanced CD8^+^ T cell effector function and promoted better tumor control by modulating differential expression of NFAT isoforms, which favored the T cell differentiation into effector cells and inhibited their terminal differentiation into exhausted cells (233).

Despite these promising results in preclinical models, little is known about the long-term effects of these treatments in human cancers. There are several ongoing clinical trials investigating epigenetic modifier drugs such as EZH2 (enhancer of zeste homologue 2), LSD1 (lysine-specific histone demethylase 1A), C9a (histone-lysine N-methyltransferase EHMT2) and BET (bromodomain and extra-terminal) inhibitors in combination with ICB or other immunotherapies (207, 234) (**Table 2**). Results from these trials are eagerly awaited to clarify the effects of these drugs beyond preclinical models and might establish them as a new class of anticancer drugs to be used in clinics.

## Concluding Remarks and Future Perspectives

IFN-I and IFN-III signaling exert potent antitumor effects through the induction of an antiviral state within tumor cells, characterized by tumor cytostasis and apoptosis. These responses are crucial in mediating benefits to well-established therapeutic approaches, such as radiotherapy, chemotherapy and ICB-based immunotherapies. As such, the activation of these pathways in the TME may be used as a biomarker of therapeutic response and prognosis.

Also, strategies aiming to enhance antiviral responses in the TME have been exploited in preclinical models and clinical trials either as adjuvants or single agents to potentiate anti-tumor immune responses, with the aim of converting poorly immunogenic and refractory tumors into highly immune infiltrated tumors and boosting multiple therapeutic approaches. These strategies include (i) promotion of immunogenic cell death (e.g.: through oncolytic viruses and cytotoxic therapies, such as low dose radiotherapy and chemotherapy); (ii) direct activation of innate immunity sensors (e.g.: STING agonists) and (iii) re-expression of EREs through epigenetic modulation (e.g.: HDACi and DHA).

Therefore, IFN-I and III responses hold promising translational potential either as biomarkers or therapeutic targets in oncology, and this field might be revolutionized in the next few years by the approval of therapies capable of modulating antiviral responses that can cross-react with tumor cells, pointing out to their great potential to enhancing antitumor immune responses and overcome resistance to different therapeutic approaches.

## Author Contributions

GV: Conceptualization, writing, review, editing and figure design. WF: Conceptualization, writing, review and editing. VC: Writing, review and editing. TM: Conceptualization, supervision, writing, review and editing. All authors contributed to the article and approved the submitted version.

## Funding

This work was supported by funds from the São Paulo Research Foundation (FAPESP) to GV (fellowship 2020/10299-7) and TM (grant 2018/14034-8), by grants from the National Institute of Science and Technology in Oncogenomics and Therapeutic Innovation (INCITO) funded by FAPESP (grant 2014/50943-1), and the National Council for Scientific and Technological Development (CNPq, grant 465682/2014-6).

## Conflict of Interest

The authors declare that the research was conducted in the absence of any commercial or financial relationships that could be construed as a potential conflict of interest.

## Publisher’s Note

All claims expressed in this article are solely those of the authors and do not necessarily represent those of their affiliated organizations, or those of the publisher, the editors and the reviewers. Any product that may be evaluated in this article, or claim that may be made by its manufacturer, is not guaranteed or endorsed by the publisher.
